# Obtaining Granules from Waste Tannery Shavings and Mineral Additives by Wet Pulp Granulation

**DOI:** 10.3390/molecules25225419

**Published:** 2020-11-19

**Authors:** Katarzyna Ławińska, Szymon Szufa, Remigiusz Modrzewski, Andrzej Obraniak, Tomasz Wężyk, Andrzej Rostocki, Tomasz P. Olejnik

**Affiliations:** 1 Łukasiewicz Research Network-Institute of Leather Industry, Zgierska 73, 91-462 Lodz, Poland; k.lawinska@ips.lodz.pl (K.Ł.); t.wezyk@ips.lodz.pl (T.W.); a.rostocki@ips.lodz.pl (A.R.); 2 Faculty of Process and Environmental Engineering, Lodz University of Technology, Wolczanska 213, 90-924 Lodz, Poland; remigiusz.modrzewski@p.lodz.pl (R.M.); andrzej.obraniak@p.lodz.pl (A.O.); 3 Faculty of Biotechnology and Food Science, Lodz University of Technology, Wolczanska 171/173, 90-924 Lodz, Poland; tomasz.olejnik@p.lodz.pl

**Keywords:** shavings, tanning waste, agglomerates, disc granulation

## Abstract

This paper presents the results of research on the granulation process of leather industry waste, i.e., tanning shavings. It is economically justified to granulate this waste together with mineral additives that are useful in the processes of their further processing. Unfortunately, the granulation of raw, unsorted shavings does not obtain desired results due to their unusual properties. In this study, the possibilities of agglomeration of this waste were examined by a new method consisting of the production and then the granulation of wet pulp. During granulation, no additional binding liquid is added to the granulated bed. As part of this work, the specific surface of granulated shavings, the granulometric composition of the obtained agglomerates, and their strength parameters were determined. The use of a vibrating disc granulator, the addition of a water glass solution (in the pulp), dolomite, and gypsum made it possible to obtain durable, mechanically stable granules.

## 1. Introduction

The industrial sector, including tanneries, distilleries, and paper and textile industries, plays an essential role in the manufacturing of various products to fulfill the needs of society. These industries continue to emit waste contaminants to the environment during the manufacturing process [1]. The tannery industry is one of the most polluting industries considering the generation of a massive amount of liquid and solid waste [2,3]. The literature indicates that normally only 255 kg of finished leather is produced by processing 1 ton of raw hides, where approximately 75% of the raw material is discarded in the form of collagen and flashing wastes [4]. In their work, the authors Mella et al. [5] have shown that the processing of one metric ton of rawhide results in 200 kg of tanned leather, 190–350 kg of non-tanned waste, and 200–250 kg of tanned leather waste. The most concerning organic waste includes that rich in lipids, cellulose, and protein, with a high organic load and poorly biodegradable compounds added due to tanning technologies [6,7].

The main form of solid waste is leather shavings and the hides and sludge that flow into wastewater treatment plants [8,9]. Shavings comprise a waste product remaining after the tanning process itself, which makes them resistant to biological degradation as they comprise a material that is hard to dispose of in any manner whatsoever. Chromium is widely used in leather tanning. Large amounts of gaseous, liquid, and solid waste containing chromium compounds are discharged into the air, surface water, and soil, causing their contamination. By using a hyphenated HPLC-ICP MS (High-performance Liquid Chromatography coupled to Inductively Coupled Plasma Mass Spectrometry) technique, Leśniewksa et al. [10] define the chromium species released during alkaline extraction of various soils collected from a contaminated area of an old tannery. In an analysis of the composition of tannery solid waste, anaerobic digestion has also been suggested as a potential alternative treatment [11]. Agustini et al. developed a biogas production method of solid waste originating from a tannery that uses chromium salts as a tanning agent [12]. Verma et al. presented a comprehensive view of the microbial and functional diversity associated with solid tannery waste. The presence of vast bacterial amounts suggested that these microbes might be contributing towards the biodegradation of pollutants present in the tannery waste dumpsites [13]. Resource utilization of organic matter in tannery sludge has drawn great attention [14]. The paper by Ma et al. [15] explores a greener alternative for the removal and recovery of chromium from tannery sludge (contaminated by a high concentration of Cr and refractory organics) by an ultrasound-assisted supercritical water oxidation approach. In further work, Pietrelli et al. [16] developed a most convenient approach to deal with these powders, generated as a by-product when chromium-containing tannery waste is treated with plasma pyrolysis and managed to recover the heavy metals they contain. The paper by Luu [17] explores tannery wastewater treatment after the activated sludge process digestion was carried out using electrochemical oxidation by SnO_2_/Ti and PbO_2_/Ti inactive anodes.

About 60% of chemical industrial products are produced in granular form [18,19]. Methods of non-pressure [20] and pressure granulation are often applied to process by-production waste [21]. In his work, Wzorek [22] developed a technology of sewage sludge fuel production consisting of the initial mixing of the components and the subsequent proportions, and simultaneous granulation and drying in a drum granulator. Ghasemi et al. made compost granules with a high bulk density through the drum granulation method using sugar beet molasses as the binder [23,24]. Greinert et al. [25] demonstrated the process of ash granulation with the mineral addition of bentonite. One factor initiating the wet granulation process is the provision of combining liquid to the bed [26].

Drum [27], disc [28,29] and vibrating [30] granulators are well known. At the same time, during disc or drum granulation, apart from the agglomeration process, an impact effect of granules takes place, leading to the disintegration of the newly formed granules. The destructive impact effect of the granulated raw material slows down the kinetics of the process, increasing the unit energy inputs [31,32,33]. The work of the disc granulator consists of rotating the disc, inclined at an angle of 40–60 degrees, around its axis, which is mounted on a disc rigidly mounted on a rotating drive shaft. As a result of the rotation of the disc under the influence of frictional forces, inertia, and gravity, the granular material placed in it circulates and is granulated as a result of adding the moistening liquid. In the vibration method, the bed is set in motion necessary for its granulation by means of an inclined vibrating chute. However, the solutions indicated have drawbacks. The granulator disc is stuck inside by the moist bed, while high-performance processes cannot be carried out in a vibrating granulator. The solution to the problem may be a device whose design combines the two ideas described above. The vibrating disc granulator is a specific combination of the traditional method of disc granulation with vibration granulation. The advantages of both methods are manifested in this device. The disc vibrations, for example, reduce the unfavorable phenomenon of the granulated material particles sticking to the surface of the disc, and also facilitate the binding of the liquid fed through the spray nozzles with the granulate. There are known methods of agglomeration of tannery shavings with the use of pressure-less disc agglomeration [34]. These methods consist of dosing the granulator with the shavings and mineral material, their initial mixing during the rotation of the disc, and then moistening the mixture with an aqueous solution of water glass using nozzles. This publication describes the possibility of segregating fine-grained shavings and mineral material during the rotation of the disc. This segregation is due to the different moisture and bulk densities of both components of the mixture and may result in their separate granulation after moistening with a water glass solution.

Tannery shavings have irregular, flocculent shapes, which is why they tend to form larger agglomerates, which, in turn, hinder operations such as screening, dosing, pouring, and loading into containers. The solution to these problems would be to give shavings regular shapes, and thus, obtain a loose particulate deposit. This can be ensured by processes using granulation. Agglomerates of granulated shavings with the addition of relevant mineral substances may then be used for the production of leather or leather-like materials, composition leather, composite materials based on fragmented collagen fibers, fertilizers, fibrous materials, and protein hydrolysates used, above all, for shaping seed-grains.

The aim of this work is to present a new method of obtaining durable and mechanically resistant tannery shaving granules with the same content of mineral additives in the entire volume of the bed and uniform distribution of granulate grain size.

## 2. Results

After the granulometric analysis of the produced agglomerates on the control sieves, the strength parameters of individual fractions were tested. The analysis concerning the value of compressive force at which the destruction of granules occurred was conducted on the Instron analyzer, which measured the value of force in the function of the displacement of a head that compressed a granule. Each time resistance of five granules from each size class was examined, and then the arithmetic mean was calculated. As a result of the tests, eight granulated products were obtained in the agglomeration processes, 60 granules were selected from the granulated product obtained in each of the tests that were then dropped onto a concrete floor from a height of 1 m, and the number of non-broken granules was counted [35] for the purpose of establishing their breaking resistance.

### Tests of Manufactured Granulates

The composition of the individual fractions of granulated strings by the wet pulp method is presented in Table 1.

The surface area parameter is directly related to the number and size of the pores in the material. Pores can have different dimensions, shapes, and volumes and can form a variety of channel systems. Due to the size and the adsorption phenomena occurring in them, according to the IUPAC (International Union of Pure and Applied Chemistry) classification [36], pores are divided into macropores with a diameter above 50 nm, mesopores with a diameter of 2–50 nm, and micropores with a diameter below 2 nm.

**Figure 1:**
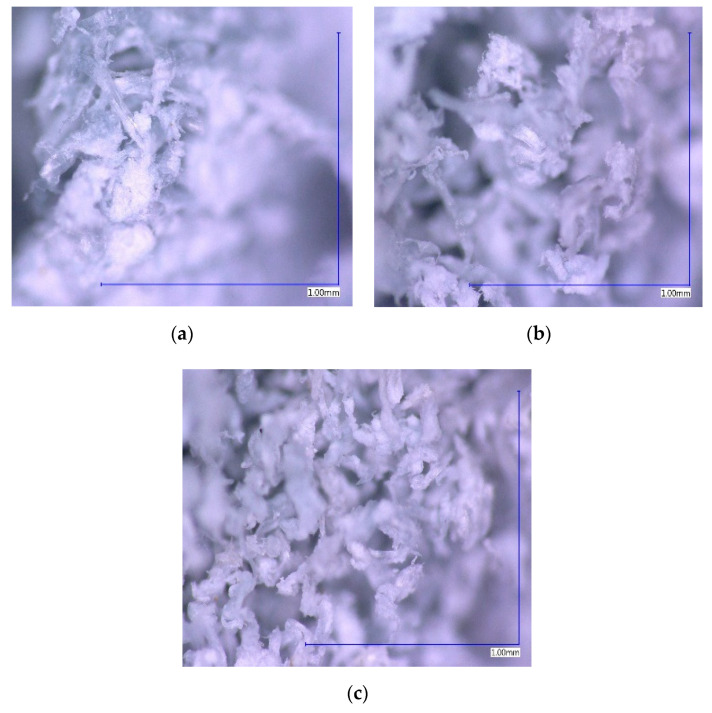
Structure of tanning shavings ((**a**)—C1, (**b**)—C5, and (**c**)—C6 fractions).

The pore structure of the tested tanning shaving samples consists mainly of mesopores and macropores (Table 2). This is evidenced by the relatively low BET (Brunauer–Emmett–Teller specific surface area (SSA), which was in the range of 2.55–3.51 m^2^/g. Comparing the tested samples to natural meso and macropore materials, such as carbonate rocks and dolomites, tanning shavings have a specific surface that is similar to them. BET space of calcium carbonate (CaCO_3_), sodium bicarbonate (NaHCO_3_) or hydrated lime (Ca(OH)_2_), used e.g., in the power industry, amounts to several m^2^/g. Appropriate mechanical and thermal activation only allows an increase in the value of their surface properties to several m^2^/g [37,38]. The structure of the shavings subjected to granulation processes is presented in Figure 1 (magnification × 200).

## 3. Discussion

**Figure 2:**
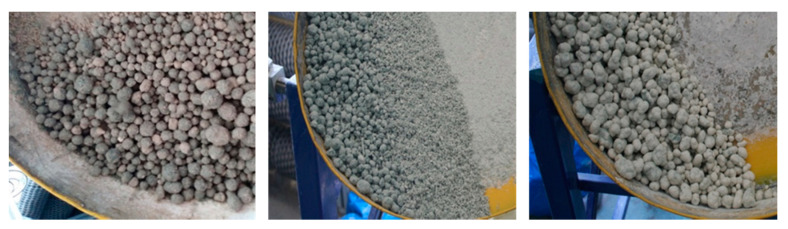
The process of granulation of tanning shavings in pulp according to methods 2, 4, and 7.

Tannery shavings consist of a material that is difficult to screen, but after ensuring appropriate conditions for the process, sieve classification is possible. These conditions are, above all, a sufficiently long screening time and the application of a very thin layer of material to the screen. The optimal time for screening raw tanning shavings is 20 min. It should be emphasized that after sieving the coarse fraction (the longest fibers) on a 2.0 mm sieve, the further sieving process on a 1.0 mm sieve was no problem and required a shorter time. It is also worth noting that the content of the fraction intended for granulation (0–2.0 mm) is in total almost 60% by weight, so only about 40% of the starting material requires grinding or other management. Based on the results of the granulation of classified and non-classified tannery shavings, it was found advisable to carry out an earlier screening process of this waste. An example of the methods of pulp granulation is shown in Figure 2.

**Figure 3:**
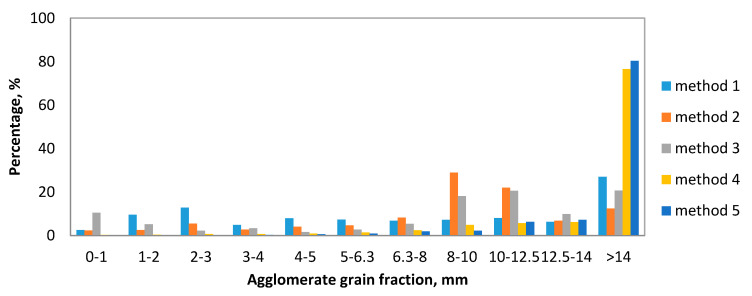
Granulate grain-size (methods 1–5).

**Figure 4:**
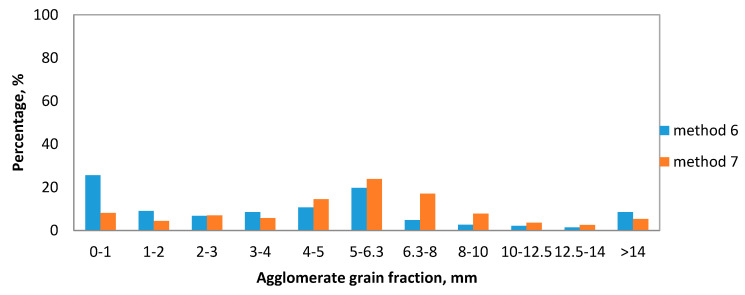
Granulate grain-size (methods 6–7).

The analysis of the granulometric compositions (Figure 3) of the obtained granulates made of the 0–2 mm fraction of strings (C3, C4, C5) shows that in methods four and five, the agglomerates obtained were found to have the highest percentage of grains with the largest fraction (>14 mm). In these tests, the mass mineral addition (dolomite) was the highest. A typical calcium oxide-based sorbent studied in the literature is dolomite [39,40]. As a result of the granulation carried out according to the second method, the granulate produced contained the highest percentage of the fractions of 8–10 mm and 10–12.5 mm. This method uses the addition of dry and wet gypsum. The main component of phosphogypsum and natural gypsum is calcium sulfate dihydrate (CaSO_4_ × 2H_2_O) [41]. Granules obtained by methods six and seven (C4, C5 fraction, i.e., 0–1 mm) were characterized by a different composition (Figure 4), as their composition was dominated by small fractions. The granulometric composition of the resulting agglomerates depends on the fraction of the shavings subjected to the wet pulp granulation processes. Granules obtained according to methods two, four, and seven are shown in Figure 4.

**Figure 5:**
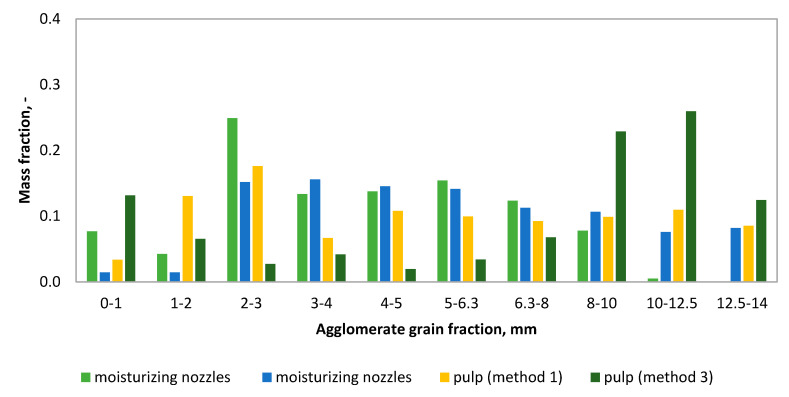
Comparison of disc granulation methods with the addition of wet gypsum.

**Figure 6:**
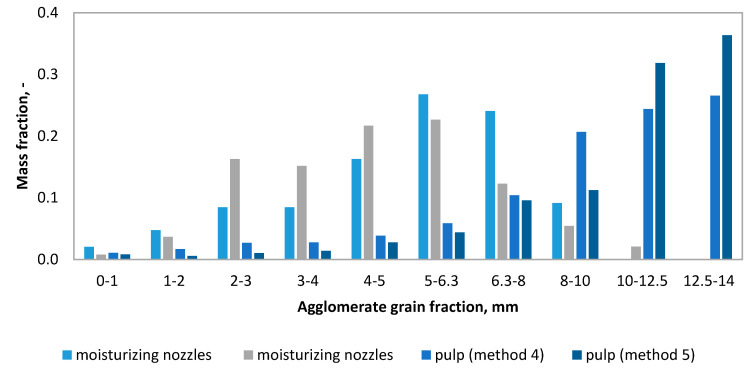
Comparison of disc granulation methods with the addition of dolomite.

The mass fractions of the individual fractions of the granules obtained were also analyzed in relation to the traditional disc granulation, in which the moisturizing liquid was delivered to the bed by means of a hydraulic nozzle in the form of drops with a diameter of 0.01–0.5 mm under a pressure of 30 kPa [34]. The comparison was made for the granulation methods of 0–2 mm shavings with the use of the same mineral additives (wet gypsum, Figure 5, and dolomite, Figure 6). Comparing the two methods of disc granulation, significant differences can be noticed in the mass fraction of the individual fractions of granules obtained from tanning shavings and mineral additives. As a result of granulation of the strings by the wet pulp method, both with additions in the form of wet gypsum and dolomite, granules of larger diameters were obtained.

The results of the endurance tests are presented in Table 3 and Table 4. The highest values of the average force breaking the granules of individual fractions were obtained by methods four and five with the highest mass addition of dolomite. High values were also obtained for methods one and two with the highest mass addition of the aqueous composition solution. In these cases, the resistance of the granules to discharge was also the highest. The breakage of granules has consequences in the processing, transport, and final use of granular products [18]. The guild granules produced as part of the work are more resistant to discharge than fly ash granules from coal combustion [42] with the addition of water glass and bentonite solutions produced by the disc granulation method [43]. The strength parameters of the granules are strongly influenced by the addition of water glass solution. Soluble sodium silicate binders (water glass) have been widely used in industries due to their low cost, odorless nature, and non-toxicity. They also offer a higher level of binding performance such as strength, durability, and water resistance [44]. In a further study [45], the authors investigated the possibility of using a sodium silicate solution in a drum granulation process for the production of bio-waste granules. Compost was successfully densified into good-quality spherical granules for the purpose of convenient storage and transportation. The process of biomass compaction depends on many factors related to the material and process [46,47]. The agglomeration process of waste materials favors their storage (increase in bulk density) [48]. The literature indicates that powders exhibit auto-granulation behavior under mechanical vibration (e.g., titania powder). The equilibrium size of the granules produced an increase with increasing vibrational intensity. This suggests that higher energy or power of vibration creates granules with stronger structures [49].

The potential application area of the granulate [50] produced involves the production of composite materials based on comminuted collagen fibers and agricultural technology. The test results indicate extensive application possibilities for the composites based on crushed collagen fibers and mineral additives. One of these possibilities concerns footwear insoles, where quality is an important element determining the hygienic characteristics of shoes. Additionally, the properties of these obtained composites can be individually shaped according to the demand and application through the selection of process parameters. Young’s modulus values classify these composites in the group of polymers (EVA) and certain materials from the group of elastomers; rigid polymer foam [51,52,53], for example. Collagen hydrolysate from leather waste contains specific amino acids that are nutrients and irrigation agents for plants. The compatibility of protein hydrolysate with many other chemical materials (bentonite, dolomite, kaolin) increased absorption at leaf level, and biodegradability paves the way for toxicity reduction and plant health stimulation [54,55,56,57]. Protein hydrolyzes from tanning waste can induce plant defense responses and increase the plant’s tolerance to various abiotic stresses (salinity, drought, temperature, and oxidizing conditions) [58,59]. The investigation in this article constitutes a multi-stage cycle of research in the field of recycling and reusing waste generated by tanneries.

## 4. Materials and Methods

### 4.1. Leather Shavings

Tanning shavings are characterized by a very low bulk density, which for dry material does not exceed 0.1 g/cm^3^. Therefore, they occupy a large volume and, moreover, have irregular, flock-like shapes, which cause their spontaneous formation into larger agglomerates. In the process of traditional granulation, they do not succumb to good moistening with a binding liquid and are not effectively combined with other additives, for example mineral powders, which are necessary at a later stage of their processing. The mineral additives and the shavings are then granulated separately, obtaining a product with a very diversified, uneven composition. It has also been found that such granules have low mechanical strength. The instability of the granulate obtained makes it difficult or impossible to carry out such operations such as screening, dosing, pouring, or loading into hoppers. Waste shavings from chrome leather treatment technology were used for this research. Their granulometric composition was determined using sieve analysis.

### 4.2. Examination of the Specific Surface of the Shavings

As part of this work, porosimetry tests were carried out with the use of low-pressure gas adsorption of samples of tanning shavings. The structural studies were performed on the ASAP 2020 volumetric adsorption analyzer (Micromeritics Instruments Corporation, Norcross, GA, USA). The measurement was carried out using the low-pressure nitrogen adsorption method (N_2_) at a temperature of 77 K and in the range of absolute pressure 0–0.05 MPa, corresponding to a relative pressure of 0 < *p*/*p*_0_ ≤ 0.5. Before the measurement, each sample was prepared by degassing in a vacuum of 10–3 mmHg at 393K for 1 h. On the basis of the equilibrium sorption points of nitrogen adsorption, the parameters of the maximum sorption capacity and specific surface area were determined. The Brunauer, Emmett, and Teller (BET) model in the relative pressure range of 0.05 < *p*/*p*_0_ ≤ 0.30 was used to characterize pores filled in a multilayer manner. This model is based on the adsorption isotherm, which was determined on the basis of the adsorption points in accordance with Equation (1):

(1) $$a_{BET}\left(P\right)=\frac{a_{mBET}C\frac{p}{p_{0}}}{\left(1-\frac{p}{p_{0}}\right)\left[1+\left(C-1\right)\frac{p}{p_{0}}\right]}$$

where: $a_{BET}\left(P\right)$ [cm^3^/g] is the sorption equilibrium point, $a_{mBET}$ [cm^3^/g] is the total sorption capacity, C [−] is the adsorption equilibrium constant depending on the difference between the heat of adsorption for the first layer and the heat of condensation. The BET specific surface area was determined according to Equation (2):

(2) $$SSA=a_{m}\omega N_{A}$$

where: $SSA\;\left[m^{2}/g\right]$ is the specific surface, $\omega \;\left[{\mathrm{nm}}^{2}\right]$ is the surface occupied by a single adsorbate molecule in the monomolecular layer, the so-called the sitting area, and $N_{A}\;\left[{\mathrm{mol}}^{-1}\right]$ is Avogadro’s number.

### 4.3. Methods of Granulation of Shavings

**Figure 7:**
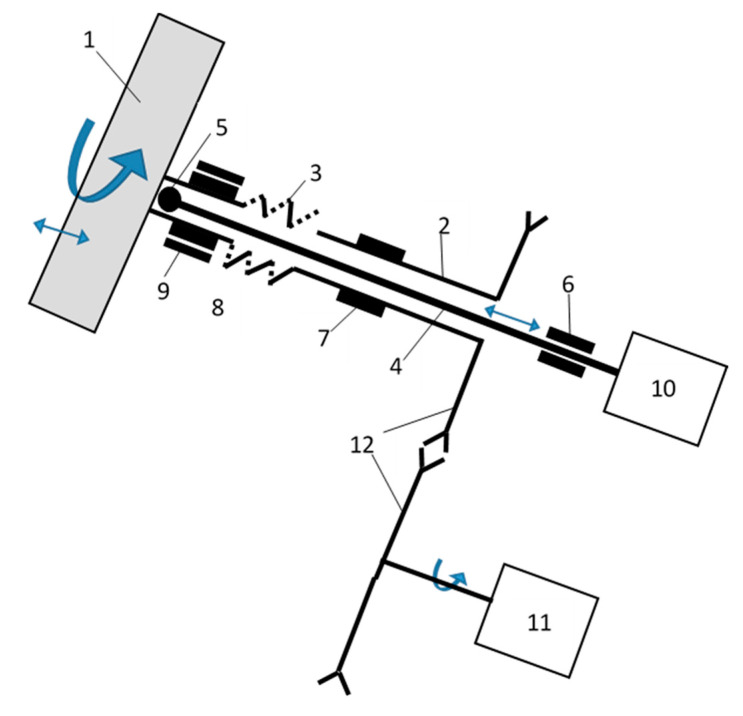
Vibrating disc granulator.

The tannery shaving granulation process was carried out on a vibrating disc granulator shown in Figure 7. This granulator is equipped with a rotary plate (1) mounted on a hollow shaft (2), which is equipped with an elastic element (3). The spring element enables the reciprocating movement of the plate and the upper part of the shaft on which the plate is mounted. Inside this shaft is a rigid rod (4), which vibrates in the axial direction. Rectilinear, axial vibrations of the rod are forced by an electro vibrator (10). These vibrations are transferred to the granulator disc by the pusher (5) located in the axis of rotation of the disc inside the hollow shaft (2). The pusher can be connected to the plate by means of a rotary bearing (the plate performs a rotary movement, the pusher does not rotate), or it can freely slide on the plate surface. The pusher rod (4) is mounted in a slide bearing (6), enabling reciprocating movement in the axial direction of the rod. The lower part of the hollow shaft (2) is mounted on the rotating bearing (7) because it only performs the rotational movement provided by the electric motor (11) through belt (12) or gear transmission. The upper part of the shaft (2) is mounted in a rotary bearing (8), the housing of which is inside the second slide bearing (9). Such a mounting enables simultaneous rotary (inner bearing) and reciprocating (outer bearing) movement of the upper part of the shaft and the granulator disc. The granulation process was carried out on a granulator plate with a diameter of 0.55 m, inclined at an angle of 45° at a rotational speed of 30 rpm, with a vibration frequency of 50 Hz and an amplitude of 1 mm.

Obtaining the material with the highest possible moisture content is a key issue in the further stage of processing the shavings, which is the granulation process. On the other hand, an excess of binding liquid may cause it to drip onto the surface of the granulator disc and interfere with the granulation process. The ideal degree of moisture of the material (pulp) before granulation is the dynamic moisture coefficient (i.e., the moisture content remaining in the material despite the inertial forces (driving forces of the process, e.g., vibratory screening)) on the material. In the research, it was obtained by soaking 150 g of classified tannery shavings (fractions 0–1 and 0–2 mm) each time in a 75% solution of sodium water glass R-145, and then placing them on a vibrating sieve to drain its excess. In the next step, the mixture (pulp) was placed on the granulator plate. After 3 min, mineral additives, dolomite, and dry and wet gypsum were added to the rotating bed. No binding liquid was added in the granulation process. Granulation was run until the majority of the fine-grained material was attached to the granules, making sure that the agglomeration of granules did not occur (no coalescence phenomenon). After completion of the granulation process, the granulate produced was dried for 48 h at 75 °C, and then its particle size composition was determined, and selected strength parameters were determined. Granulation methods were performed according to the trials presented in Table 5.

## 5. Conclusions

As part of the work, granules were produced by the method of wet pulp granulation from waste classified tanning shavings, and then their selected parameters were determined. The presented method of granulation is a free-flowing agglomerated granular bed containing both mineral and organic ingredients, which is easy to store, transport, and dose. Moistening the bed with a water glass solution provides agglomerates with high mechanical strength. The introduction of a mineral additive to the mixer facilitates the subsequent agglomeration of fine-grained fractions of the shavings, which, together with the drying process, provides dry granules on the outer surface, which form a non-caking bed of flowability, allowing for free transport of agglomerates for subsequent technological operations.

## 6. Patents

Method of producing agglomerate from tanning shavings: P425268, P425277, P425287, P425288 (polish patents). Vibrating disc granulator: P435768 (polish patent).

## Figures and Tables

**Table 1:** Grain-size composition of shavings.

Sample	Fraction, mm	Mass Fraction %
C1	>4	17.5
C2	2–4	23
C3	1–2	25.5
C4	0.5–1	20
C5	<0.5	14

**Table 2:** Grain-size composition of shavings.

Sample of Shavings	Total Sorption Capacity, cm^3^/g STP	Specific Surface, BET (Brunauer–Emmett–Teller) m^2^/g	Adsorption Equilibrium Constant	Correlation Coefficient
C1	0.81	3.51 ± 0.02	12.97	0.999
C2	0.59	2.55 ± 0.02	20.72	0.999
C3	0.65	2.84 ± 0.02	19.13	0.999
C4	0.61	2.66 ± 0.01	23.67	0.999
C5	0.60	2.63 ± 0.01	23.85	0.999

**Table 3:** Average force destroying granules.

Agglomerate Grain Fraction	Average Force Destroying Granules N						
	Method 1	Method 2	Method 3	Method 4	Method 5	Method 6	Method 7
0–1	1.83	3.5	1.3	2.9	3.5	2.7	0.5
1–2	3.46	5.6	4.8	6.1	8.3	4.1	0.9
2–3	4.07	6.8	5.2	25.1	21.2	5	1.2
3–4	4.34	7.8	6.2	30.6	29.1	5.2	1.6
4–5	6.65	9.7	7.7	60.2	67.2	6.6	2.2
5–6.3	7.02	23.1	8.4	82.5	73.5	7.1	2.7
6.3–8	18.34	29.8	11.6	129.8	126	13.8	4.4
8–10	39.97	31.5	26	158.8	185.4	27.8	10.3
10–12.5	43.99	40.2	28.6	190.9	198.7	28.8	13.1
12.5–14	65.438	41.9	34.9	200	200	35.9	17.1
>14	149.838	95.4	96.3	200	200	48.1	26.2

**Table 4:** Number of non-broken granules.

Agglomerate Grain Fraction	Number of Non-Broken Granules, pc						
	Method 1	Method 2	Method 3	Method 4	Method 5	Method 6	Method 7
5–6.3	60	60	56	59	60	55	54
6.3–8	59	60	57	60	60	59	59
8–10	59	60	59	58	60	58	59
10–12.5	59	60	58	59	59	59	58
12.5–14	60	60	57	60	59	58	54
>14	60	60	57	60	58	60	58
sum	57/60	60/60	44/60	56/60	56/60	49/60	42/60

**Table 5:** Process parameters of granulation.

Method	Fraction of Shavings [mm]	Water Glass Solution [g]	Mineral Additive	Mass of Mineral Additive [g]	Time of Granulation [min]
1	0–2	1000	Wet gypsum	500	10
2	0–2	1000	Wet gypsum Wet gypsum	500 285	10
3	0–2	800	Wet gypsum	500	10
4	0–2	800	Dolomite	1700	13
5	0–2	600	Dolomite	1500	8
6	0–1	800	Wet gypsum Dolomite	400 950	15
7	0–1	800	Wet gypsum Dolomite	400 1250	18
